# Advancements in functional smart and wearable textiles for sportswear applications

**DOI:** 10.1039/d5ra07231j

**Published:** 2025-12-19

**Authors:** Md Touhidul Islam, Md Imran Hosen, Tarikul Islam, Md. Abdullah Al Mamun, Tariful Islam

**Affiliations:** a Department of Textile Engineering, Mawlana Bhashani Science and Technology University Santosh Tangail 1902 Bangladesh; b Department of Textile Engineering, University of Scholars Dhaka 1213 Bangladesh; c Department of Textiles, Merchandising, and Interiors, University of Georgia Athens GA 30602 USA tarikul@uga.edu; d Department of Textile Engineering, Jashore University of Science and Technology Jashore 7408 Bangladesh; e Department of Textile Engineering, Donghua University Shanghai 201620 China

## Abstract

Growing demand for smart textiles and wearable technologies, in the sports sector, for enhancing performance as well as comfort, motivated researchers to go further in the past decade. Recent advancements and integration of these technologies in sports have boosted athletic performance and comfort, which are explored in this study. Sports textiles have evolved rapidly, commencing with the usage of natural fibers, which then progressed with the introduction of synthetic materials and smart technologies. These remarkable improvements have led to major developments in thermoregulation, moisture management, and overall comfort of sportswear. In addition, the combination of sensors and electronic circuits built into textile materials also enables the real-time measurement of physiological parameters, including ones such as heart rate, body temperature, and muscle effort. Our investigation illustrates the importance of smart textiles, nanotextiles, and wearable electronics in performance enhancement, particularly in competitive sports, where high-tech textiles are increasingly used to gain a competitive edge. This study concludes with the discussion of the challenges facing smart textiles at the development and application phase, such as durability, washability, energy source, and sustainability, while identifying potential prospects for future research and development.

## Abstract 2

Growing demand for smart textiles and wearable technologies, in the sports sector, for enhancing performance as well as comfort, motivated researchers to go further in the past decade.

## Graphical abstract

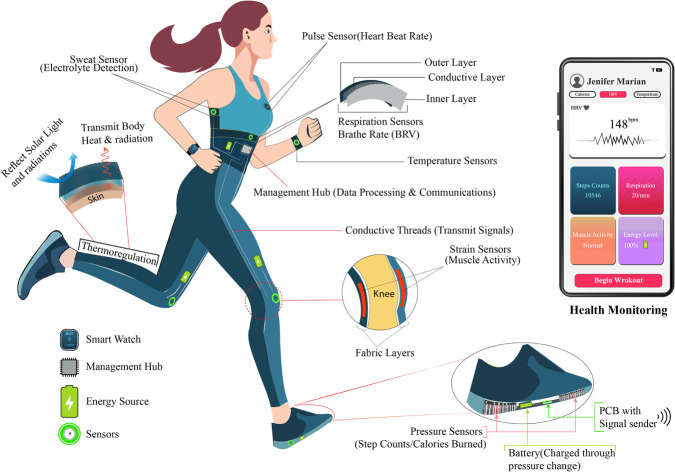

## 1. Introduction

Originally, sportswear was designed for athletic activities. Over time, it has changed to include style, comfort, and performance. In the early stages, sportswear was mostly about function, which meant helping athletes move freely and meet their physical needs. Today, sportswear is as much about style and lifestyle as it is about performance. Modern athletes, especially in track and swimming, rely on high-performance apparel made from advanced fabrics and fibers. The evolution of sportswear began with visionary designers like John Redfern. In the 1870s, he created some of the first specialized outfits for women in sports such as tennis, horse riding, yachting, and archery.^1^ The design work by Redfern put comfort and ease of movement first, which were important for sports and being outside. This was a significant shift from the restrictive clothing of the era, allowing for greater participation in sports.^2^

The international textile industry has been significantly impacted by the growth of sportswear and activewear markets. Sportswear worn as an everyday fashion item to specialized gear for individual sports is available in the market. Only about 25% of sportswear is thought to be used for exercise or competitive sports, but the amount is higher at present. High standards of comfort and care-free design are required by consumers for all garment categories. Keeping the wearer comfortable in terms of thermoregulation can improve performance in sportswear. This was accomplished by creating clothing that aids in preserving body temperature and moisture output at or near typical levels. New fibers, yarns, structures, and coatings have been created and released onto the market in recent years for the sports and functional textile markets. In addition to the materials already recognized, novel possibilities for new functional textiles are provided by microfibers derived from various polymers. Furthermore, the potential to finish fibers with features like temperature storage, medicine delivery, or anti-microbial behavior opens new possibilities. To meet the demands of sportswear and numerous other uses, special high-performance fibers need to possess a variety of qualities.^3^

Globally, there has been a noteworthy surge in engagement in physical activities since the 1980s, and leisure clothing has been impacted by performance sportswear. With apparel accounting for 72% of sales compared to 28% for footwear, in 2018, the UK sportswear sector was estimated at around £5.5 billion.^4^ The UK sportswear industry is divided into items used for sports and activity purposes and those for fashion and leisure wear. This sector accounts for approximately 14.6% of all clothing and footwear purchased in the UK.^5^ Sales worldwide are accounted for by the top 10 major sportswear markets, which are as follows: the United States at 35%, China at 10%, Japan at 7%, Brazil at 5%, Germany at 4%, France at 4%, the United Kingdom at 4%, Italy at 3%, Russia at 3%, and Spain at 2%. However, the market development estimation for outdoor sportswear in China and India between 2012 and 2017 is 142% and 205%, respectively.^6^ The market size of smart textiles, expected to surpass USD 5.55 billion by 2025, and has the healthcare and satisfaction sectors as major drivers, indicates the growing importance of technology.

Over the next five years, the telemedicine segment based on wearable sensors is anticipated to grow at a rate faster than 50% CAGR (compound annual growth rate). As more “E-textiles”—intelligent, flexible integrated systems with wireless communication, sensing, and actuator capabilities—are developed, research into the possibilities presented by modifying textile materials at the nanoscale for the creation of new smart, adaptive and active functionalities continues to grow. These wearables and high-tech fabrics are examples of E-textiles. A complicated range of multidisciplinary issues in material design, hierarchical integration, control schemes, and manufacturing are involved in the construction of these systems.^7^ Three categories for sportswear are commonly found: performance wear, outdoor wear, and sports-inspired clothing. A marked increase in the overlap between sporting and leisurewear in the last twenty years has occurred due to increased demand for both indoor and outdoor activities. This was encouraged, among others, by sociocultural changes such as the lower availability of leisure time, greater consciousness of health, development of sport infrastructure, and presence of attractive and practical sports clothes. Using modern textile technologies, manufacturers of sportswear respond to different consumers and market needs by offering products made of performance fabrics.^6^ Smart textiles have embedded sensors that detect and respond to environmental stimuli like mechanical, thermal, or chemical changes, enhancing functionality for the wearer. They can be classified as passive, active, or ultra-smart. Passive smart textiles do not interact with their environment or change the measured variable. E-textiles are a broad category of smart textiles that contain electronic components. In addition to functions in numerous additional end-uses, smart textiles can be built with applications in climate management, sportswear, health monitoring, and enhanced comfort.^8^ Electronic elements are woven into smart textiles, such as conductive yarns, actuators, and sensors, giving them the ability to recognize, respond to, and change with their surroundings. These textiles are useful in healthcare for patient monitoring and rehabilitation since they can track physiological signals, including movement, body temperature, and heart rate. Smart wearables improve lifestyle optimization and personal health management by offering real-time data and connectivity. In addition to the medical field, wearables find use in the sports, military, and entertainment sectors for communication, immersive experiences, and performance monitoring. In the playground, wearable technology and smart textiles are transforming the way athletes engage with their surroundings and providing fresh opportunities for performance improvement, health tracking, and connectivity. Smart textiles provide real-time feedback, whereas conventional sport fabrics do not serve the purposes. However, they involve higher production complexity and cost. Unlike passive performance fabrics, active and ultra-smart textiles introduce electronic dependency, which may limit washability and mass adoption. As a result of developments in material science, electronics, and data analytics, their integration into daily life is expanding.

Unlike textile fabrics, sportswear textiles are purposefully engineered for performance enhancement, utilizing specialized polymers, smart technologies, and nanomaterials.^9^ Sportswear textiles include multiple performance functions in one material, including dynamic moisture management, real-time physiological monitoring, and thermal regulation.^10^ These fabrics are characterized adaptive response to variations in body and environmental situations, incorporating electronic sensing functionalities while preserving comfort and flexibility. The multifunctional design, developed across the fiber, yarn, and fabric levels, enables garments to sustain prolonged athletic performance without compromising efficiency or wearability.^11^ Their development necessitates a multidisciplinary approach, drawing upon materials science, microelectronics, and advanced textile engineering. Smart fabrics represent an advanced progression by embedding sensors and adaptive systems that enable garments to react immediately to shifts in physiological and environmental conditions, thereby evolving performance apparel into an interactive and responsive platform.

Smart textiles are often characterized as passive, active, or ultra-smart based on their interaction with stimuli. Passive smart textiles sense environmental changes but do not respond or modify the measured variables; examples include moisture-sensitive fabrics that indicate sweat levels or UV-sensitive materials that change color in sunlight.^12^ Active smart textiles are designed to respond dynamically to internal or external stimuli. For example, shape-memory fibers can adjust compression in response to body movements. Similarly, thermo-responsive fabrics regulate insulation according to temperature fluctuations.^13^ Ultra-smart textiles combine sensing, actuation, and data processing, enabling real-time monitoring and autonomous response. For instance, garments with embedded microcontrollers can detect heart rate, modify thermal insulation, and transfer data to mobile devices concurrently.^14^

Smart textiles have found applications across diverse sectors. In healthcare, wearable textiles monitor physiological signals such as body temperature, heart rate, and movement, allowing patient monitoring and rehabilitation. In the sports sector, smart garments deliver real-time feedback on performance metrics, including muscle activity and sweat management. Military applications include uniforms that adapt to temperature or environmental stress. In the entertainment sector, haptic feedback suits enhance immersive gaming or virtual reality experiences. The integration of electronic elements such as conductive yarns, actuators, and sensors empowers textiles to detect, respond, and adapt to environmental and user-specific stimuli, thereby boosting comfort, performance, and safety across varied applications.^15^

Electronic elements such as conductive yarns, actuators, and sensors are woven into smart textiles, enabling them to detect, respond, and adapt to environmental and user-specific stimuli. In healthcare, smart wearables like Hexoskin shirts and Sensoria socks detect physiological signals such as heart rate, breathing, movement, and gait, helping patient monitoring, rehabilitation, and injury prevention.^16^ In sports, devices such as Catapult trackers and Under Armor recovery garments provide real-time feedback on athlete performance, including fatigue levels, body temperature, and biomechanics.^17^ The military uses wearable sensor systems to monitor troop vitals and environmental circumstances, while adaptive uniforms with in-built thermal regulation boost operational efficiency.^18^ In entertainment, haptic systems like the Teslasuit deliver immersive virtual reality experiences through motion sensing and tactile feedback. Beyond sensor integration, advancements in data analytics and artificial intelligence have greatly expanded the capabilities of smart textiles. These systems can assess physiological and environmental data in real time, provide predictive health monitoring, and autonomously modify clothing qualities such as thermal insulation or compression. These combined capabilities enable lifestyle optimization, personal health management, and performance monitoring across healthcare, sports, military, and recreational applications.^19^

Advances in materials science, electronics, and data analytics have brought smart textiles into everyday life, especially in sports and healthcare.^20,21^ Despite these advancements, several challenges require careful consideration to ensure that smart textiles function reliably, remain safe for users, and are environmentally sustainable. Washability and durability are key concerns. Repeated washing can destroy electronic components and decrease sensor function.^22^ User safety is equally critical, as these textiles must adhere to low-voltage standards, remain compatible with skin, and reduce the risk of electrical hazards.^23^ Collaboration across disciplines is another challenge. Designers, material scientists, and electronics engineers need to cooperate closely to insert smart components without compromising the textile qualities.^24^ Environmental impact cannot be overlooked either, since traditional textile and electronics manufacturing consume enormous resources and generate pollution. Actionable strategies include developing modular and washable electronic components, implementing safety standards, fostering cross-disciplinary collaboration, and prioritizing recyclable and eco-friendly materials throughout the design and manufacturing process.^14^ Smart textiles need to interact with traditional textiles and the market appears to have significant unrealized potential. Instead of waiting for small, gradual adjustments, the field should push for a major, revolutionary shift.^7^

## 2. Evolution of sports textiles

### 2.1 Historical perspective on sports textiles

For centuries, textiles have played a key role in shaping footwear, especially in women's fashion shoes.^25^ The mid-19^th^ century saw the emergence of “sneakers,” which brought fabrics into athletic and everyday shoes.^26^ According to Coye, the book Strange Company by James Greenwood (1873) contains one of the earliest mentions of sneakers as canvas-topped shoes with Indian-rubber soles.^27^ Although initially produced in limited numbers, these gymnasium shoes laid the foundation for the modern athletic footwear industry. Despite this intriguing fact, the sports shoe designs found in the late nineteenth century, such as these, are more artifacts than evidence of a significant movement. Although there was a limited amount of production of these shoes, cotton canvas uppers with rubber soles were marketed as “gymnasium shoes” during the latter part of the nineteenth century and well into the twentieth. It required the rise of the middle class and the rapid expansion of recreational pursuits in the early 1900s to generate a mass market demand for athletic shoes, particularly those with textile uppers designed for basketball play. By the 60s and 70s, European brands like Adidas and Puma shifted to leather and synthetic materials. The fitness boom of the 1970s fueled demand for athletic shoes, establishing major brands like Nike and Reebok, with textiles driving the evolution of the industry.

Since the fitness boom, sports shoe manufacturers have increasingly relied on textiles for innovation. Textiles were still essential to the development of the industry. According to ‘Sporting Goods Intelligence’ latest estimates, the wholesale market for branded athletic footwear was valued at over $8.25 billion in 2003 and was expected to reach over $9 billion in 2004.^28^ Between 2004 and 2012, a massive industrial advancement in the textile economy progressed in the sector. Fabrics with special features, such as moisture-wicking, UV protection, as well as synthetic materials, likely polyester and nylon, started gaining popularity.^29^ These athletes and fitness fans had their needs met by innovations that provided improved breathability, durability, and comfort. Companies such as Nike, Adidas, and Puma are at the forefront of these advancements as they have continued to redefine the capabilities of sports textiles.^30^ Consequently, the last ten years have seen rapid growth in the sports textiles market because of increased interest in activewear as well as enhanced technology. The growth of the market between 2013 and 2023 was rapid and was sustained by a number of factors and trends that increased earnings.^31^

### 2.2 Key milestones in the development of sports textiles

**Fig. 1:**
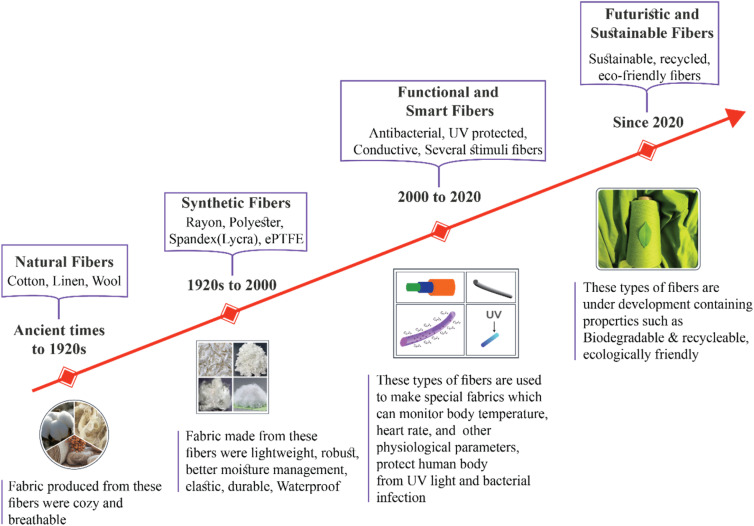
Historical development of fibers (created with Illustrator).

Sports clothing has come a long way. Synthetic fibers like polyester and nylon appeared in the mid-20^th^ century. To improve athlete performance, they provided improved breathability, durability, and transpiration. By the 1970s and 1980s, fabrics with UV protection and quick-drying properties arrived, further transforming performance wear. These innovations met the growing need for clothing that could handle long periods of activity without sacrificing comfort or effectiveness. The 21^st^ century brought the era of smart textiles, turning what once seemed like science fiction into reality. Thanks to tiny sensors and advanced materials, our clothes can now act as personal coaches and health monitors. These textiles have sensors built in to track vital signs, including muscle contraction, body temperature, and heart rate in real time. Smart textiles keep athletes comfortable and flexible while reducing the risk of injury. They also help athletes perform at their best by providing the right data at the right moment. This technology turns traditional sportswear into a responsive, interactive system. It enables on-body data processing, delivering instant feedback while keeping data secure. This integration transforms sportswear into a fully responsive and interactive system, allowing on-body data processing, also known as edge computing. It delivers instant feedback while keeping the data safe. Advances in bioengineering, materials science, and a focus on athlete needs such as safety, comfort, and hygiene have driven these innovations. Together, these advancements have created a new generation of high-performance textiles that adapt to the needs of modern athletes. Key turning points in the evolution of fibers from early practical fabrics to contemporary intelligent sports textiles are (see Fig. 1).

#### 2.2.1 Early origins: basic clothing and natural fibers

In ancient times, natural materials such as cotton, wool, and linen were used to make early sportswear. Although these textiles were cozy and breathable, they lacked the durability and moisture-wicking qualities required for high-intensity sports.^32^ During the 19^th^ century, the Textile industry saw tremendous breakthroughs due to the industrial revolution. Particularly in games like football and cricket, where having a unique team color became decisive, sports uniforms grew increasingly standard. Sportswear became more comfortable and of higher quality because of improved weaving techniques, yet wool and cotton remained the leading materials.^6^ Even though the materials of this era had limited usefulness, they set the groundwork for our understanding of mobility, comfort, and breathability—all of which were later improved by synthetic fibers. The concept of functional fabrics in sportswear began as the emphasis progressively moved from simple protection to performance.

#### 2.2.2 Creation of synthetic fibers

Between the 1920s and 1930s, the first synthetic textile, rayon, was introduced, offering a substitute for natural fibers. Compared to wool, it was lighter and had superior moisture-management qualities. Sports textiles underwent a revolution with the introduction of polyester in the 1950s and nylon in 1935. These synthetic fibers were resilient to bending and shrinking, lightweight, and robust. Because of the elasticity and durability of nylon, it was first used in sportswear as stockings and quickly spread to other sportswear items.^33^ Superior strength, flexibility, and moisture control were made possible with the introduction of synthetic fibers, which marked the first real functional advancement in sportswear. The development of intelligent and sensitive textiles in later decades was made possible by this period, which showed how material innovation might directly affect athletic performance.

#### 2.2.3 Customized apparel and enhancement of performance

During the decade of 1970s, special apparel that improved athletic performance came into being. Brands such as Adidas and Nike started emphasizing the development of breathable, lightweight textiles. The discovery of spandex, also referred to as Lycra or elastane, in the late 1950s and its use in the 1970s made it possible to design clothing with exceptional elasticity and a snug fit, which was advantageous for sports like track and field and gymnastics.^34^ In the 1980s, synthetic microfibers gained popularity, and textiles with improved moisture-wicking qualities were created. Because these fibers were thinner than natural fibers, they were better at wicking sweat from the skin and keeping sportsmen dry and comfortable. During this time, branded sportswear also gained popularity, and sports icons like Michael Jordan shaped worldwide trends in the garment industry.^33,35^ These developments reflected the transition from generic to performance-tailored apparel. The integration of spandex and microfibers finally gave the designer the chance to develop sport-specific textiles that met specific biomechanical demands, transforming passive coverings into active contributors to athletic performance, a required precursor for smart and adaptive sportswear.

#### 2.2.4 Innovative technology and smart textiles

The 1990s sports textiles, which are waterproof and breathable, made possible by Gore-Tex in the 1970s. Since they provided weather protection while letting moisture vapor escape, Gore-Tex and related materials had established themselves as industry standards by the 1990s in outdoor sportswear.^36^ In the 2000s, advances such as antimicrobial treatments, which helped sportswear stay odor-free and bacteria-free shifted the focus towards enhancing fabric utility. More textiles, including UV protection, give athletes more protection when they are outside.^37^ In the 2010s, the use of smart fabrics started to grow. Electronic components like sensors and conductive threads are included in these textiles. To optimize training and performance, smart textiles can track the body temperature of an athlete, together with heart rate, and other physiological parameters. Several companies investigated these technologies and included them in their product lines, such as Nike and Under Armor.^38^ It was during this phase that the evolution from functional to intelligent sportswear became more pronounced. The fusion of materials science and digital technology was thus worked out through the integration of membranes, antimicrobial coatings, and embedded electronics. In this way, textiles turned into interactive systems that can sense, adapt, and enhance the comfort and performance of athletes in real time.

#### 2.2.5 Future prospects for eco-friendly athletic textiles

Since 2010, the emphasis on sustainable sports textiles has been sparked by increased awareness of environmental issues. This covers the creation of biodegradable materials, recycled polyester, and ecologically friendly dyeing and finishing techniques. Companies are using these materials more frequently in response to consumer demand for more environmentally friendly goods. Future wearable technology and smart fabrics that adjust to the demands of the wearer or the environment will see a greater integration of smart technologies into sports textiles. Furthermore, improvements targeted at lessening the environmental impact of sportswear production and disposal will keep sustainability as a primary focus.^39^ The current approach places a strong emphasis on striking a balance between sustainability and functionality. Future smart textiles are anticipated to combine eco-friendly production practices with self-regulating performance features, resulting in adaptable clothing that not only responds to physiological cues but also supports environmental stewardship. This represents the next frontier in intelligent sportswear design.

## 3. Smart textiles in sports

### 3.1 Overview of smart textiles and their applications in sports

**Fig. 2:**
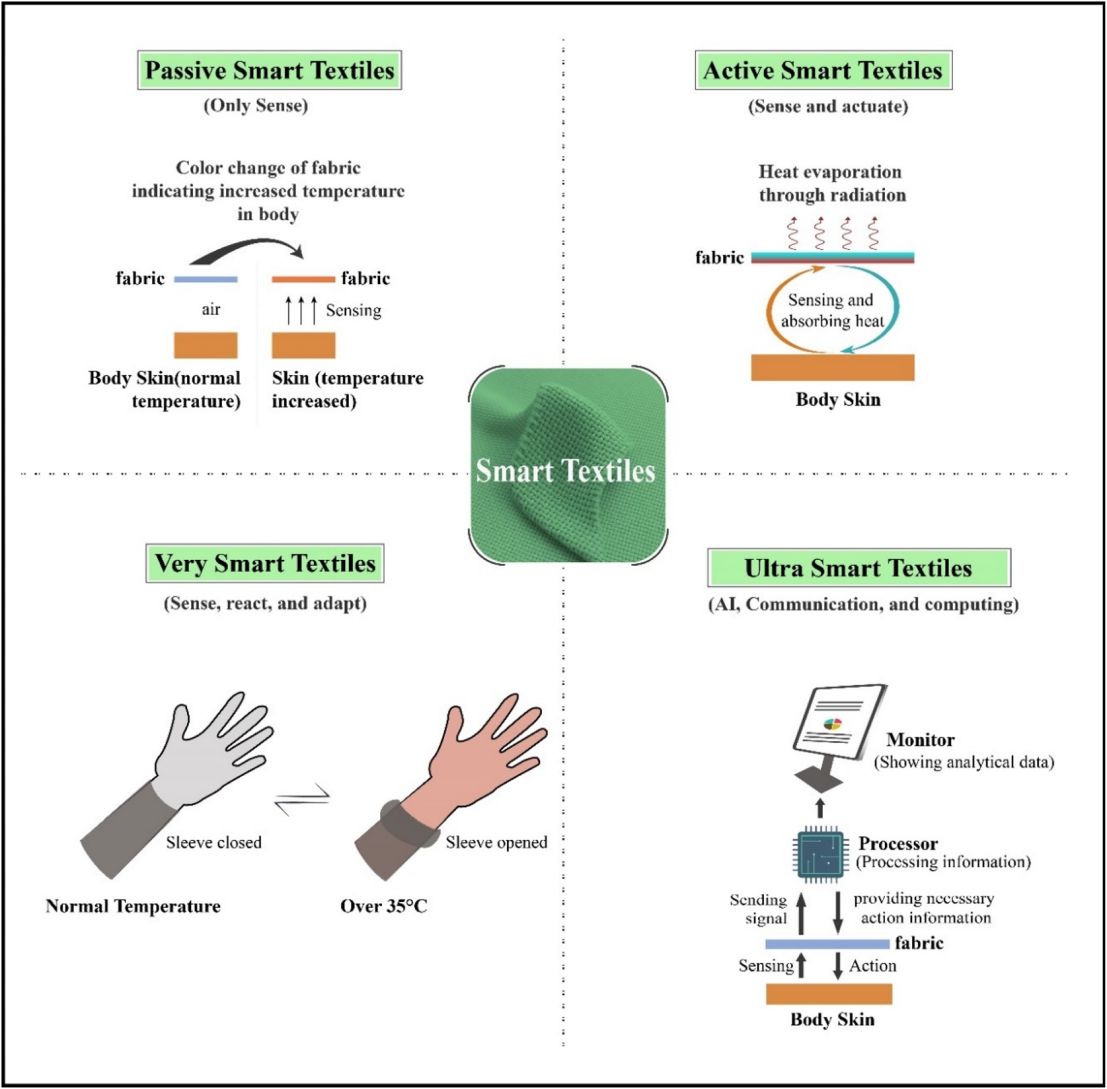
Categories of smart textiles (passive, active, very smart, advanced smart). Published under the CC-BY license^42^ Copyright 2025, The authors. Published by Springer Nature.

Many types of intelligent structures can be found in the natural world. Artificial intelligence involves developing structures that can sense and respond to external stimuli. The complexity and capability of smart clothing systems are on the rise, and these materials can be classified into four categories based on how they respond: passive smart materials only sense the stimuli or conditions in the environment (step count, calories burned, or heart rate, may be detected by passive smart clothing); active smart materials sense and respond to the conditions or stimuli; very smart and ultra-smart materials sense, react and adapt accordingly (see Fig. 2). Higher levels of intelligence can be reached from those intelligent materials that can respond or activate to perform a function in a manual or programmed manner.^40^ As a result, these materials may include three different parts: controlling units, actuators, and sensors. To provide a nervous system for signal detection, sensors are necessary in a passive smart material. Actuators are key components of active smart materials, working either directly from the detected signal or through a central control unit. At the top of the scale, in the area of extremely intelligent or smart materials, still another type of unit is critical—one that functions similarly to the brain in terms of cognition, reasoning, and capacity activation—for various applications pertaining to the human body that require electronics in textiles, which has given rise to the new field of E-textiles research.^41^

Technological developments like the use of conductive yarns have made it possible to create novel soft textile interfaces with great user acceptance.^43^ This development represents a significant leap forward in technology, opening an extensive range of wearable E-textiles with several uses, including data transfer, individual environment control, communication capabilities, and body function sensing and monitoring.^44^ Textiles can be easily formed around three-dimensional surfaces, like our bodies, because they are flexible. The development of new electrically conductive textiles for stretchy electronic systems that can be twisted or curved around intricate curves is being documented in a research paper by Christian Dils *et al.*^45^ The medical, sports and fitness, military, fashion, automotive, aerospace, environment, and energy industries are just a few of those industries that can benefit from the sensing, adapting, and responding, multi-functionality, low energy, small size and weight, ease of forming, and low-cost characteristics of smart textiles.

**Fig. 3:**
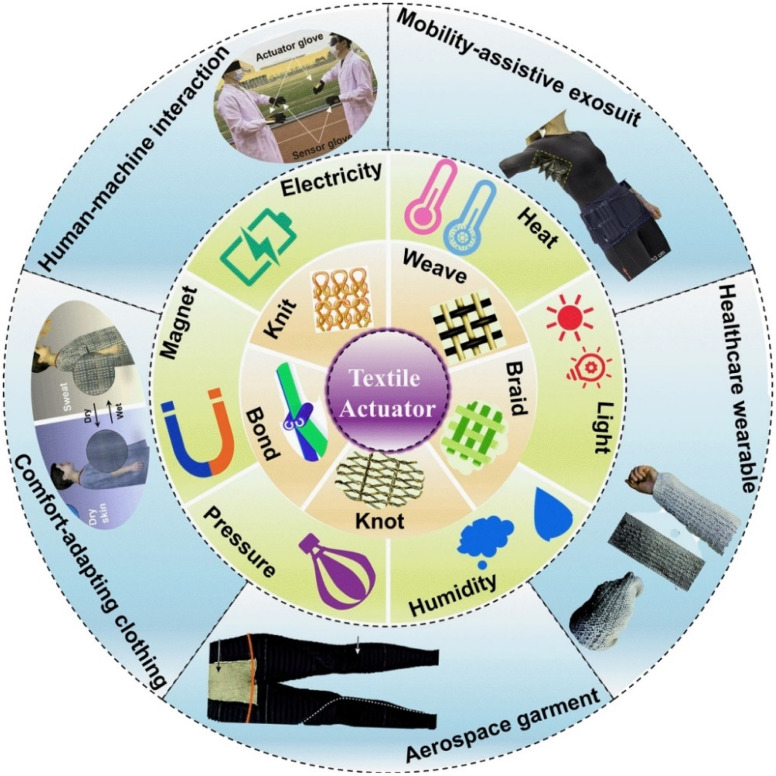
Overview of flexible textile actuators in smart wearables. Published under the CC-BY license^49^ Copyright 2023, The authors. Published by Springer Nature.

In addition to redefining material science design and engineering and advancing life quality and the environment, the study and development of these novel and valuable materials transcend scientific bounds.^7^ However, as an alternative to uncomfortable, connected systems, sensorized clothing has been developed in recent decades as a result of the developments in textiles and sensor technology. The development of sportswear has given rise to novel textile compositions tailored to certain sports for enhanced aerodynamics, polymers, and aerogels that react to stimuli, and customized coatings for managing perspiration and heat.^46^ Additionally, these specialty textiles have been combined with smaller-sized sensors to create “smart clothing”. As physiological (heart rate, and respiration), performance (posture, and movement), and environmental (temperature, and humidity) data are collected in real time, smart clothing enables athletes to play their sports without hindrance. Even stretching is possible in smart textiles nowadays. Stretch sensors, for example, are elastic bands with soft capacitors that, when stretched, yield accurate motion data about the human body.^47^ Integrated microcomputers in ultra-smart clothing enable them to perceive and intelligently elaborate a variety of data types, enabling them to anticipate future events and adapt to external demands through actuators (see Fig. 3). For instance, depending on the surrounding temperature, space suits have the ability to thermo-regulate the human body.^48^

### 3.2 Technological advancements in smart textiles

Technology is getting increasingly integrated into our daily lives, both enhancing and lessening the pressures of modern existence in different ways. To meet these needs, the textile industry offers enormous potential to improve the performance and usefulness of textiles. Smart nano textiles are going to change everything—from the materials used in industry to the garments we wear to the furniture in our homes. The upcoming revolution has raised the bar for textile performance, and there is a strong market for smart fabrics or materials with increased environmental awareness. Applications for technical and functional textiles are numerous and include anything from entertainment to security and military to individualized healthcare.^50^ Textiles are a ubiquitous and global interface, making them a perfect medium for incorporating sensors that track both the wearer and the surroundings. A flexible framework for adding sensors, monitors, and information processing devices is provided by textiles. Intelligent textiles possess the ability to perceive and respond to various environmental factors or stimuli, such as those originating from mechanical, thermal, chemical, electrical, or magnetic sources.^51^ The challenges of connectivity, bulkiness, wearability, and washability are widely documented, and a common element of smart clothing research is the overlaying of traditional electronics over a textile substrate.^52^ The creation of intelligent nano textiles has the potential to completely change how our clothes and the fibers around us work. Intelligent fabrics may now perform novel functions, including self-cleaning, sensing, actuating, and communicating, thanks to nanoscale manipulation. Innovations like carbon nanotubes, naturally conductive polymers, novel materials, fibers, and finishes, as well as antibacterial nano coatings, have all contributed to intelligent textiles.^50^ Carbon-based polymer nanocomposites can enhance the electrical conductivity, strength, and stability of E-textiles.^53^ These extra features have a wide range of uses, including in sports, fashion, healthcare, and the military. Through sensors knitted or woven into garments, smart textiles can detect vital health information about their users. Wearable shirts have been successfully implanted with fabric electrodes, which are used to monitor electrocardiograms (ECG). Long-term patient monitoring is made possible by these electrodes since the fabric electrodes record data while the electrode-infused apparel is being worn. To gather breathing signals, piezoresistive fabric sensors are knitted into shirts.^54^ These sensors can be utilized to identify movement and keep an eye on the posture and movements of the wearers. The field of medical rehabilitation could be revolutionized by wearable smart fabrics. By tracking the biometrics and movement of the patient, the sensors in the smart clothes can provide information that will help determine how beneficial the patient is. These sensors should be as seamless as possible with the clothing and should cause the least amount of discomfort possible. Sensor placement plays a role since consistent and dependable data collection is needed.^55^ A complex network of textile sensors, including conductivity, pH, salt, and sweat rate sensors combined into a lower back waistband, makes up the Boi-Tex system.^56^

A chest band is used to implement sensors for pulse oximetry, respiration, and ECG. The textile-based pH sensor employs two optical LEDs for optical detection along with a pH-sensitive dye to detect pH levels between 4 and 8.

Representative material platforms for smart textiles are described in Table 1, highlighting both their advantages and disadvantages. Conductive textiles, such as polyaniline-coated fabrics, exhibit flexibility and electrical stability for sensing and heating, though their wash durability remains a key drawback. Graphene-based infrared textiles provide adaptive heat regulation *via* tunable emissivity; nonetheless, their efficacy in the mid-infrared range remains limited. Multifunctional smart fabrics, exemplified by Smart-NT (smart nonwoven textile), combine protection, environmental sensing, and health monitoring while simultaneously reducing body temperature, but they require careful trade-offs between comfort, integration, and durability. Piezoelectric polymers like PVDF (polyvinylidene fluoride) provide energy harvesting from body motion, valued for their flexibility, though low output and reduced stability over time hinder large-scale use. In comparison, graphene systems offer superior thermal control over PVDF-based energy harvesting textiles, but they are less flexible. These differences show how material preference is determined by the intended sports use (thermal regulation against physiological sensing). Wearable biochemical sensors integrated into fabrics provide real-time monitoring of sweat metabolites, presenting significant promise for personalized treatment; however, they necessitate effective power management and data processing. Shape memory alloys, particularly NiTi-based systems, demonstrate thermo-responsive shape recovery for adaptive garment fitting, though their limited strain capacity restricts garment versatility.

**Table 1:** Summarizes the types of materials, their sensing/actuating functions, and performance metrics

Material type	Specific example	Functionality (sensing/heating/*etc.*)	Key properties (conductivity, elasticity, breathability)	Advantages	Limitations	References
Conductive textiles	Polyaniline-coated textiles	Electrical sensing, heating	Flexibility, stability	Multifunctional use	Poor wash durability, rigid structure	57
Graphene infrared textiles	Graphene-enabled infrared fabrics	Thermal regulation, sensing	Tunable emissivity, adaptive insulation	Adaptive thermal management, real-time temperature sensing	Limited emissivity modulation in mid-IR	58
Smart fabrics	Smart non-woven textile (Smart-NT)	Protection, sensing, and health monitoring	Reduces skin temperature by ≈17 °C	High impact resistance with pressure sensitivity	Challenging seamless multifunction integration	59
				Temperature reduction with long-term durability	Difficult balance of comfort, durability, and performance	
Piezo	Poly (vinylidene fluoride)	Harvests body-motion energy, powers self-sustained wireless sensors	Flexibility, stretchability, permeability, and lightweight	High energy efficiency and long cycle life	Low energy output, durability issues	60
			High energy efficiency for long cycle life	Flexibility, stretchability, and lightweight for garments		
Wearable biochemical sensors	Skin-interfaced wearable sensors	Real-time continuous analysis of sweat composition	Biocompatible, flexible	Continuous, real-time physiological monitoring	Requires stable power and data management	61
		Monitors health, stress, and diet changes				
Shape memory alloy	NiTi-based shape memory alloy (SMA)	Adaptive garment fitting	Thermo-responsive, shape recovery	Dynamically conforms to unique body topography	Limited adaptability across garment types	62

The chest band has three textile electrodes and silicone cushions used to record ECG data. Additionally, a piezoresistive sensor on the chest band measures the rate of respiration by reacting to movement in the rib cage. These sensors are built on insights from two more EU-funded initiatives, Wealthy and MyHeart.^8^ Moreover, sweat analysis can reveal important information about the health and well-being of a person, and sweat pH is determined using a fabric fluidic system with a pH-sensitive dye integrated into it. A sweat rate sensor is integrated into a textile substrate to identify the start of sweating. The waistband incorporates the sensors, which are controlled by a wirelessly connected central unit. Sweat analysis using these kinds of sensors could yield useful physiological data for applications in healthcare and sports performance.^63^

**Fig. 4:**
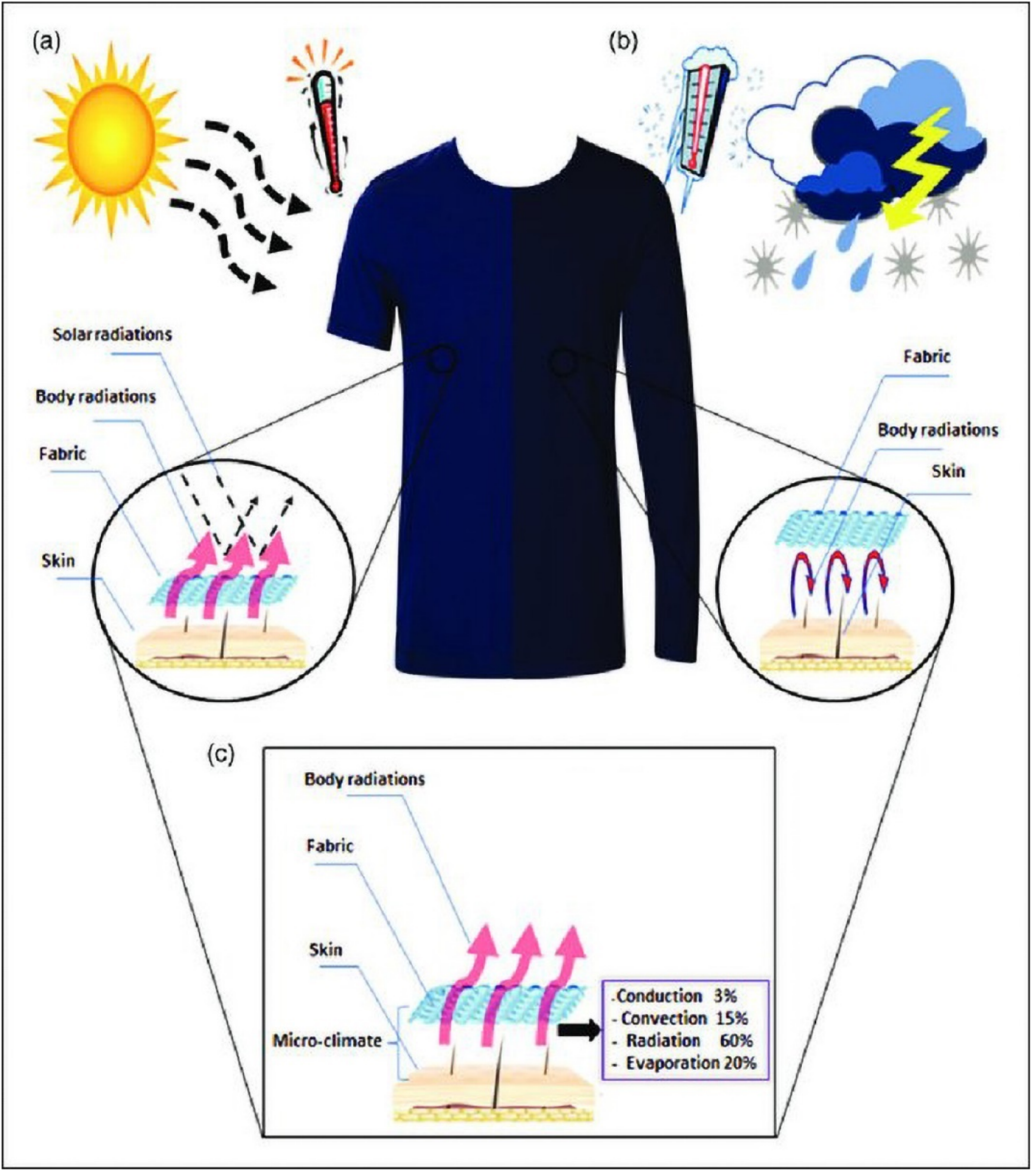
Schematic of a thermoregulating garment. (a) In hot conditions, the garment transmits body heat radiation to the outside atmosphere while reflecting solar radiation back to the surroundings. In hot conditions, this has a cooling effect. (b) A thermoregulation garment gives the wearer a warm feeling in cold conditions by reflecting the radiation from the human body. (c) The various methods that heat moves through the thermal regulating garment, such as conduction, convection, radiation, and evaporation. Published under the CC-BY license^66^ Copyright 2021, The authors. Published by SAGE.

Shape memory textiles are made of a type of material called textile memory function, which is added during the weaving or finishing process. Textiles have exceptional qualities like shape memory, high deformation recovery, good shock resistance, and flexibility under external conditions like temperature, mechanical force, light, pH value, *etc.* A “lazy shirt” was developed by the Italian business Corpo Nove. The properties of the shirt were that, when the temperature outside is high, the sleeves of the shirt will automatically roll from the wrist to the elbow in a matter of second; when the temperature decreases, the sleeves can recover and even iron themselves mechanically.^64^ The three primary categories of smart temperature control textiles are cool textiles, automatic temperature control textiles, and thermal insulation textiles (see Fig. 4). Solar thermal storage fibers and far-infrared fibers are the primary thermal insulation materials produced domestically and internationally for use in thermal insulation fabrics. Cool fabrics typically incorporate metal oxides into polyester fabrics to ensure that the interior of the garment remains cool. Metal oxide reduces the likelihood of clothing fading from light and ultraviolet rays.^64^ The ability to wear clothing that both warms them and serves as a safety signal is central for those who work in frigid climates (such as traffic police officers during the winter). To achieve a temperature-regulated smart textile that emits light and regulates temperature, it is imperative to develop smart textiles that combine thermal control with light-emitting features. One such method is coaxial electro-spinning.^65^

Fabrics with such abilities offer a wide range of development possibilities and may satisfy daily needs for raincoats and other clothing items in addition to meeting their desire to wear during activities in inclement weather, such as intense cold, rain, snow, and wind. These primarily consist of non-porous membranes, microporous membranes, intelligent waterproof and moisture-permeable fabrics, and high-density waterproof and moisture-permeable fabrics. The production of self-cleaning coatings, which can eliminate both organic and inorganic impurities *via* photocatalysis and rolling water droplets, is becoming increasingly popular. Wearable technology is essential to the production of smart textiles, and it will also help the textile industry and wearable devices to develop vertical markets. People can use these smart textiles to monitor their health and ensure the quality of their lives.^64^

An overview of the many sensor types and their useful functions found in sportswear is provided in Table 2. Clothing-integrated capacitive strain sensors quickly translate body deformation into electrical signals.^67^ Vital sign monitoring is made possible by physiological sensors, such as sweat analyzers and ECGs, which have a high-power requirement. Inertial measurement units (IMUs) are lightweight and portable, making them useful for real-time motion tracking. Fiber-based temperature sensors rely on thermochromic materials to detect body heat changes with high sensitivity, without needing extra power. Optical fiber sensors integrated into elastic belts offer highly accurate respiratory monitoring in real time with low power needs.

**Table 2:** Different sensing approaches used in sportswear

Sensor type	Principle of operation	Placement in garment	Measured parameter	Accuracy/response time	Power requirement	References
Strain sensor	Capacitive sensing	Belts, gloves, and knee protectors	Strain in body or garment	Less than 30 milliseconds	Passive	68
Wearable physiological sensors	Data sensing and AI analysis	Gadgets, accessories, or clothes	Physiological signals (HR, BP, temperature, ECG, EEG, and sweat)	83% accuracy; real-time (transmission-limited)	High power consumption	69
Wearable inertial sensors (IMU)	IMU motion sensing with data fusion	Torso, limbs, or joints	Movement techniques and kinematics	Real-time feedback	Moderate; depends on sampling and data transmission	70
Flexible fiber-based temperature sensor	Temperature-induced color change	Torso, sleeves, and chest areas	Temperature changes	High sensitivity; short response time	No external power needed	71
D-shaped plastic optical fiber (POF) sensor	Bending-based optical sensing	Elastic belt around the abdomen	Respiratory rate (RR) and breathing patterns	High accuracy (*R*^2^ = 0.9977); real-time monitoring	Low to moderate	72

### 3.3 Exploration of smart nano-textiles and their applications in sports

A lot of research has been conducted in the textile industry by the sports industry to help improve athletic performance, personal comfort, and protection from several elements. Synthetic fabrics that were previously thought to be less effective than natural fabrics now have high-performance characteristics. Numerous products that are intended to enhance wearer comfort are commercially available; examples include moisture-management textiles like cool and breathable waterproof fabrics like Gore-Tex®.

**Fig. 5:**
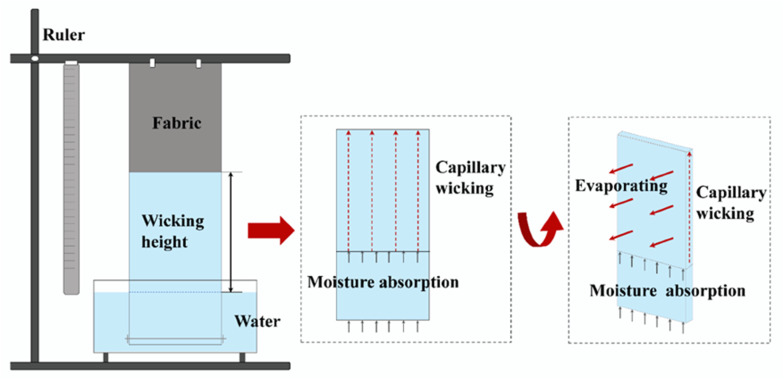
Water wicking and evaporating occur simultaneously in a typical vertical wicking test. Published by the CC-BY license^73^ Copyright 2020, The authors. Published by MDPI, Basel, Switzerland.

Sweat must be allowed to evaporate to preserve the comfort of wearer and the natural thermoregulatory function of the body. High-performance moisture-wicking fabrics worn next to the skin transfer sweat away from the body and onto the outside of the garment, where it evaporates more quickly. The wonders are accomplished by using synthetic microfibers, which act as wickers rather than absorbers of moisture, making the garments more comfortable to wear (see Fig. 5).^73^ Phase-change technology, like Outlast Adaptive Comfort®, even makes it possible to maintain a consistent body temperature. Clothing is becoming more comfortable, more suited to the wearer, and satisfying their demands thanks to the introduction of nanotextiles. The adidas_1 running shoe adapts its shock-absorbing properties to the runner's unique style, pace, body weight, and running surface using sensors, a computer, and a motor. Nike has also produced a smart running shoe with a wireless sensor that pairs with an iPod to play different playlists tailored to several types of exercise. The shoe also tracks metrics like time, distance, pace, and calories burned.^50^ Shoe-based sensors, such as those used in Nike+ systems, are simpler to calibrate but provide less full-body tracking than textile-integrated sensors. Conversely, although they are still more difficult to manufacture and power, textile-based nanosensors allow for more comprehensive physiological mapping. Several textiles are designed to enhance performance; for instance, nanotech swimsuits for Olympic swimmers were created with a biometric knitted nylon/elastane structure that mimics the ridges on shark skin^74^. Recent developments use sensing capabilities to provide real-time physiological state awareness, which provides valuable information on physical capabilities, training status, athletic potential, and responses to various training regimens. The employment of wearable sensors in the field for biochemical analysis, vital sign monitoring, and kinematic analysis is highly desired.^75^ Utilizing strain sensors derived from piezoelectric materials, biomechanical analysis can create wearable kinesthetic interfaces that can identify posture, enhance movement efficiency, and lower the risk of injury.^50^ By applying stress and strain to the fabric, these textiles can be used to measure physiological movements that put pressure or strain on the material and influence its conductivity.^76^ Wearables with conductor-loaded rubbers and piezoresistive ICPs (intrinsically conducting polymer) allow for continuous monitoring of vital signs and body kinematics. By preserving the flexible and tactile qualities of textile, this method produces genuinely wearable materials, which is an advantage. The fluidic channels of fabric have integrated sensors that track the composition of perspiration. People become more conscious of their specific healthcare demands when control electronics and wireless data transmission enable real-time signal analysis and provide users with feedback regarding their well-being.^50^

## 4. Comfort aspects of sportswear

The four categories of comfort that sportswear users perceive their comfort in are thermo-physiological, psychological, skin sensorial, and ergonomic (ease of body movement) wear comfort. Fabric breathability and moisture management properties have an impact on thermo-physiological comfort. The clothing serves as a barrier to prevent heat and vapor transfer from the skin to the surrounding environment. There are three ways that moisture can pass through the fabric: through the convection mode, which is the moving air near the skin, through the moisture sorption–desorption process on the fabric, and through diffusion, which is caused by the moisture vapor gradient across the fabric.^63^ They confirmed that the degree of physical activity significantly influences the skin temperature of athletes as well as the moisture content of the microclimate.^77^ Skin moisture levels are influenced by the moisture absorption capacity and rate of transfer of the fabric.

### 4.1 Factors affecting comfort in sportswear

#### 4.1.1 Thermal comfort

Three desired characteristics of human comfort are thermal, tactile, and psychological comfort. These are related to the movement of heat, liquid moisture, and air through textile materials, keeping the wearer dry and maintaining a consistent body temperature of 37 ± 1 °C.^78^ Surface roughness and hairiness have an impact on thermal comfort properties; rougher fabrics have a smaller skin-contact area, while more hairy fabrics, which allow for more air to circulate through the fabric, feel warmer.^79^ These factors, along with yarn spinning techniques and fabric construction variables, are major contributors to the warm-cool feeling of any fabric. Additionally, compared to spun polyester fabrics, micro-denier polyester fabric has superior heat transfer, quicker sweat evaporation, and a cool initial contact on human skin.^80^

#### 4.1.2 Moisture management

Fabrics composed of finer yarns and greater stitch lengths showed improved comfort for humid situations, as it is more permeable to air and water vapor; with the single jersey plated interlock fabrics, the thermal resistance of the fabric increased dramatically with an increase in yarn linear density.^81^ An analysis of the liquid moisture transportation characteristics of active sportswear fabrics made with polyester filament with non-circular cross sections confirms that finer filaments wicked more effectively in planes than coarser filaments, with vertical wicking also being noted. Wetting time rose, maximum wet radius dropped, rate of absorption fell, spreading speed decreased, and overall moisture management capacity decreased with the multidirectional moisture and rise in cover factor.^82^ More moisture management capacity values are found in polyester knit fabrics than in viscose and cotton fabrics.^83^ Compared to polyester, natural fibers, for example, viscose and cotton fabrics, provide better tactile comfort but inferior moisture management. In a similar vein, synthetic microfibers wick better than natural fibers, albeit at the expense of reduced sustainability and breathability, which indicates that the material choice reflects a trade-off between thermos-physiological performance.

#### 4.1.3 Influence of fabric structure and fiber blends

The moisture management properties of bi-layer knitted fabrics composed of various blends of viscose, polyester, modal, and polypropylene fibers in the outer and inner layers have been found to be significantly influenced by fabric structures, but the liquid transportation capability has declined with an increase in the tightness level.^84^ They concluded that the modal fiber of the outer layer and the micro-fiber polyester of the inner layer had superior comfort qualities and were the most appropriate for active sporting applications. Analysis of the moisture-management characteristics of wool, wool blends with cotton, and wool blended with polyester revealed that while wool/cotton-plated fabrics are good at distributing liquid in the bottom surface, knitted 100% wool fabrics performed poorly at spreading liquid on the top and bottom surfaces of the fabric.^85^ The perspiration transfer rate and perspiration spreading across the surface of the outer layer are also influenced by the yarn counts of the inner and outer layers. The more yarn counts in the inner and outer layers, the faster the spreading and the longer the wetting time.^86^

### 4.2 Advances in base fabric technologies for enhanced comfort

#### 4.2.1 Moisture management in base layers

Sportswear is indispensable for the physiological comfort of a player, especially the base layer that is worn next to the skin. The principal ability of the base layer materials to regulate moisture was made possible by the quick wicking of liquid moisture away from the skin. Base layer clothes with a tighter fit are more comfortable than ones with a slack fit.^63^ However, compared to looser constructions, tighter base layers may restrict airflow and result in heat buildup, underscoring the necessity of optimizing fit based on activity type and climate circumstances. Good thermal conductivity, moisture management, and tactile qualities are desired qualities of inner fabric layers utilized in active applications. A base layer with good thermal conductivity might increase cooling effectiveness, while improved moisture management and tactile qualities would give the wearer the required comfort.^87^ Synthetic base-layer clothing was more successful than cotton clothing at actively lowering moisture retention while preserving the ideal skin temperature. This was found from a study that examined the thermoregulatory response of the human body to base layers with hot and cold garments.^88^ The heat generated during a sporting activity needs to be simultaneously dispersed through the base layers of fabric, in order to keep the body temperature within a tolerable range.^89^ The amount of perspiration that an individual should create in an hour depends on the type of sports activity. To attain a high level of comfort, the base layer textiles should absorb heat and moisture and disperse them simultaneously. Improved moisture management qualities are obtained by combining wool with bamboo and polyester fibers.^90^

#### 4.2.2 Thermal regulation and innovative cooling textiles

**Fig. 6:**
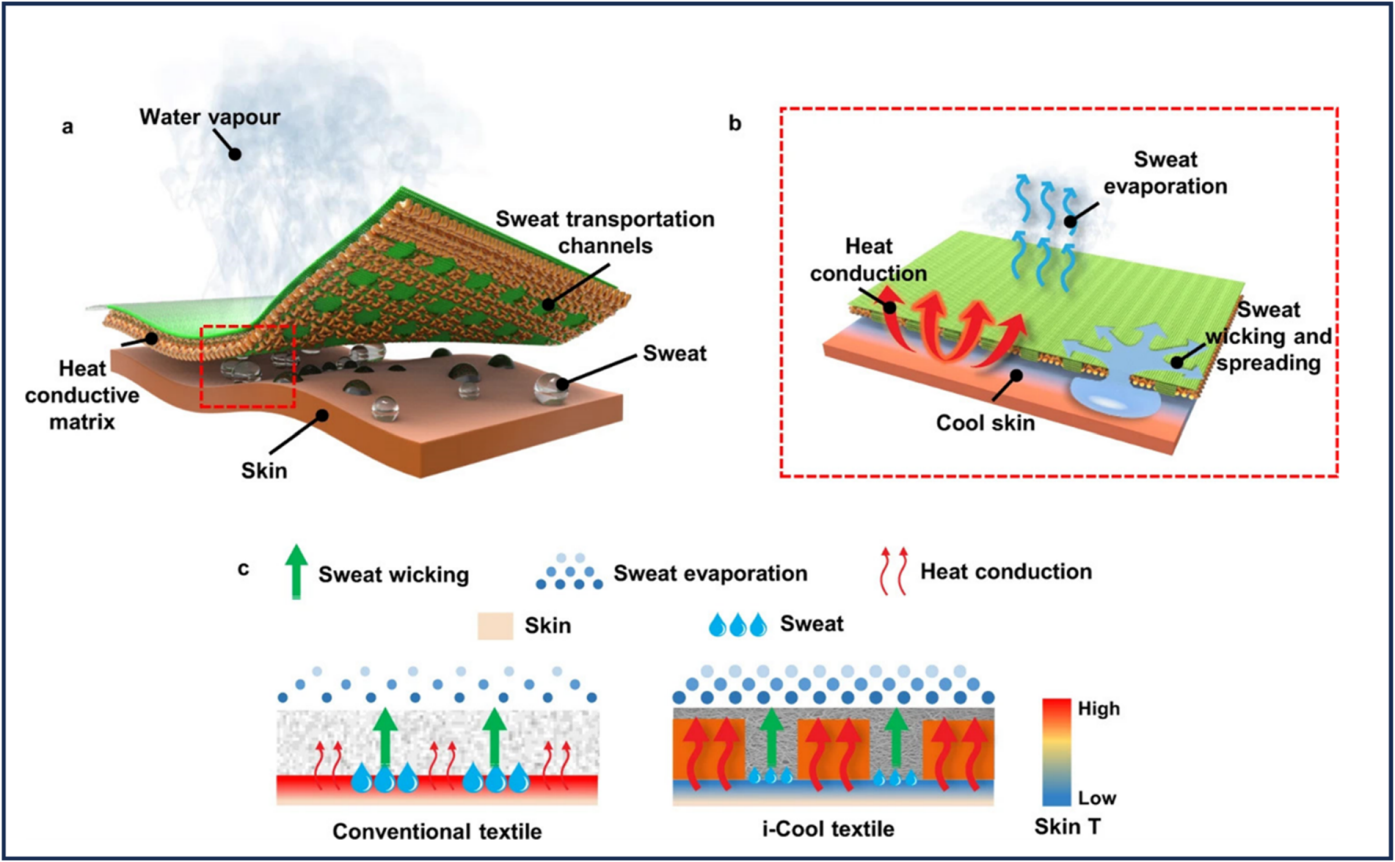
(a) Schematic of the i-Cool textile, (b) schematic of the working mechanism of the i-Cool textile, (c) comparison between conventional textiles and the i-Cool textile. Published under the CC-BY license^91^ Copyright 2021, The authors. Published by Springer Nature.

Sweat transport channels and the heat-conductive matrix work together to give textiles a solution for managing perspiration. The water transport channels in the human body can swiftly transfer sweat from the surface to the vast part of the skin. To promote rapid evaporation, the heat-conductive matrix effectively transfers body heat to the site of evaporation. In the meantime, it may effectively apply the evaporative cooling effect to human skin.^91^ To alleviate the discomfort of a wet and sticky feeling, conventional textiles often provide comfort through the buffer effect of absorbing perspiration. Its low evaporation rate and evaporative cooling efficacy, however, prevent it from effectively chilling skin and could eventually compromise the buffer effect. In contrast to regular textiles, the i-Cool textile not only transfers perspiration but also offers a superior heat conduction pathway for accelerated evaporation and removal of a significant amount (see Fig. 6). Thus, by making excellent use of perspiration, the i-Cool textile can assist the human body in achieving a greater cooling effect while significantly reducing perspiration. The difference in heat transport capabilities is depicted by the weight disparity in the red arrow drawing. In the drawing of the sweat evaporation, the dot size and density contrast illustrate the various evaporation capacities. The drop size of sweat drawing, contrast demonstrates how i-Cool cloth can lessen perspiration production.

The 50/50 blend ratio increases the rate of liquid moisture absorption to 20% and 35%, respectively. The moisture management qualities of knitted double-faced fabrics were examined, and it was discovered that the inner and outer faces of polypropylene/cotton fabric had superior moisture management qualities.^92^ For winter sportswear materials, single-knit constructions are commonly used as the foundation layer.^63^

With skin-fitting clothing, the superior softness of the single jersey textiles results in increased comfort and fewer tactile sensations. It also provides good form retention, flexibility, extensibility, and recovery. The structural form of a single jersey loose fabric has a lower liquid moisture contact angle and a higher transfer wicking ratio than that of a tight fabric. Fabric moisture management qualities and microclimate drying time are highly influenced by the kind of fiber, yarn, and fabric factors.^86^ Compression, resilience, and surface qualities of single-knit fabrics have typically deteriorated, whereas handling features, including tensile, bending, and compression properties, have improved. Cotton single jersey fabric performs better for summer innerwear, whereas cotton double pique fabric performs better for summer outerwear interference. The knitted undergarments composed of wool-acrylic blends offer a higher degree of thermal insulation than woven textiles because the knit structures trap more air.^93^

#### 4.2.3 Smart textiles and adaptive materials in sportswear

While smart textile solutions are implemented on textiles utilizing mechanical, chemical, or electrical technologies, or a combination of these, wearable computing just needs electronics. Textiles or smart fabrics are made of fibers and can respond to external stimuli and interact with their surroundings. The amalgamation of electronics and textiles offers novel methods for illumination, temperature regulation, energy conservation, interaction, detection, quantification, and oversight. Apart from electronics, smart fabric can respond to stimuli that are thermal, chemical, mechanical, or magnetic.^94^ While textiles that can change and adapt to changes, like thermochromic materials, are always a part of smart textile technology, electronics are not a necessity for such technology. The main purpose of those smart textile applications with electronics integration is to keep an eye on the environment or the user based on output data that can help, support, care for, or indirectly protect the user. Adaptive® technology of HeiQ was utilized by Bekaert Textiles. Adaptive textiles can adjust dynamically to temperature and moisture variations to provide the best possible comfort and performance.^95^ Wearable applications in sports, medicine, and healthcare frequently use smart textiles or clothing. For instance, several functions can be monitored *via* wearing textile electrodes. Applications in mHealth (mobile health) have shown wearable technology to be advantageous.^94^

## 5. Wearable technologies and sensors

### 5.1 Integration of wearable electronics in sports textiles

Textile integration of wearable computing components provides novel and widespread monitoring opportunities. The promise of providing novel and widespread monitoring opportunities is fulfilled by the combination of textile elements with wearable computing components. The domains of health, sports, and medicine, as well as protection and security, military apparel, and protection, are pertinent application fields. By the end of 2015, 76.1 million wearables had been sold globally, according to the Worldwide Quarterly Wearable Device Tracker of IDC (international data corporation). The figure includes wearables that are simple (primarily activity and fitness trackers) as well as wearables that are smart, such as smartwatches, smart textiles, and smart eyewear. In 2019, it was projected that 245 million would be sold. In comparison to 2015, that was a 300% increase. Since 2013, when sales in Europe alone reached 3 billion euros, wearable technology and apps have been among the fastest-growing sectors in the technical industry. Up to 9 billion euros is how analysts value the growth of the European market till 2018.^96^ Modern wearable technologies are becoming more powerful and complicated, which presents a significant usability difficulty. It suggests the simple use of textiles or garments while still offering a deep understanding of the human body. Developers and manufacturers must target a broad audience that can utilize the new technology because wearables are becoming ubiquitous in society.^97^ Textile-based wearables offer a more natural interface and more data accuracy than wrist-worn devices, but they have additional manufacturing and durability issues. The application area, necessary sensing precision, and user comfort all play a major role in the decision between the two.

#### 5.1.1 Physiological monitoring and sports performance

**Fig. 7:**
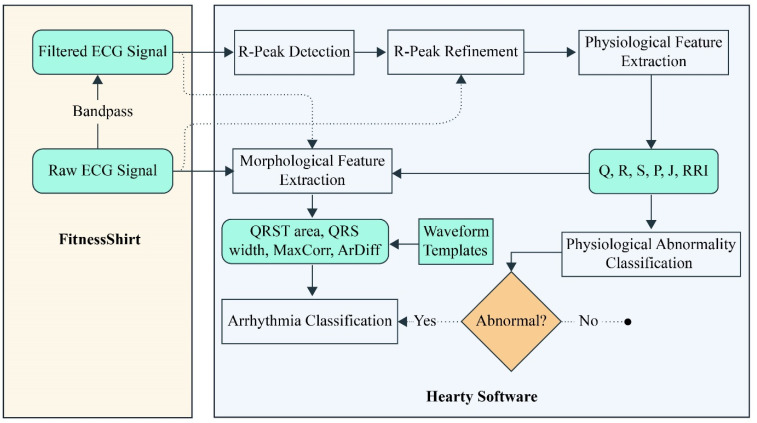
Current implementations of the signal processing and arrhythmia detection system are found in the mobile device processing software (Hearty) and FitnessShirt. In the diagram, the steps carried out in the embedded hardware are displayed on the left side. Following transmission, the signal is processed further in software on a mobile device, as seen on the right. The morphological features that are computed are the QRST (waveform) area, the QRS width, and two features that are obtained from normal beat waveform templates: the area difference (ArDiff) and the maximum correlation coefficient to the templates (MaxCorr) (created with Illustrator).

Sports have always been a vital part of human civilization. Whether it is running, leaping, or team sports, the pursuit of new human records excites millions of spectators worldwide and puts more strain on athletes and coaches. Professional athletes and trainers, therefore, always search for fresh approaches to improve their performance. Sports applications for wearables are highly promising because they are often lightweight, portable, and tiny. The real-time position-tracking technology of Red FIR and the Fitness Shirt together allowed us to measure vital signs like breathing and ECG in addition to position data from whole ice hockey or soccer games.^98^ The analysis of the heart rate is called heart rate, or HRV (heart rate variability) analysis. HRV is used in a variety of fields, including sports and training sciences, arrhythmia diagnosis, and sudden cardiac death. FitnessShirt with hearty software is designed to filter and process them to detect R-peaks and key waveform features (QRS, P, T, *etc.*). These features are analyzed for morphological and physiological patterns to classify arrhythmias and detect abnormalities (see Fig. 7). Hearty Software performs real-time classification for health monitoring.^99^

#### 5.1.2 Sensor technologies for health and performance monitoring

Some research proposed utilizing only the respiratory rate interval (RRI) signal for the early diagnosis of arrhythmias. The identification of patients, or medical conditions of athletes, may be improved by such early detection. One of these studies solely considered the previous individual RRI when computing features addressing the oscillation of RRI caused by respiration (respiratory sinus arrhythmia). With a naïve Bayes classifier, they were able to achieve mean class-dependent classification accuracies of 90%. The requirement for this method was that the aberrant heartbeat was preceded by regular heartbeats. Refining the R-peaks discovered by any QRS (Q wave, R wave, and S wave) detection method is required to obtain the most accurate estimate of the genuine HRV. To determine the true R-peaks (in ECG signal processing, R peak identification is essential for identifying and diagnosing cardiovascular illnesses, and CVDs) utilizing the filtered and raw ECG signal, they therefore created a peak refinement post-processing step.^100^ The advantage is that negative or aberrant QRS complexes, which are frequently observed in irregular heartbeats that originate in the ventricles, can also be precisely detected through wearable smart textiles. In this sense, the benefits of the integration of wearable technologies into sports textiles are unimaginable.

### 5.2 Textile sensors for performance monitoring

#### 5.2.1 Development and function of textile-based sensors

Most of the wearable sensor research so far has gone toward creating tools that gauge bodily characteristics like heart rate, respiration, and activity.^101^ In sports, textiles are frequently utilized to absorb bodily fluids and transfer them away from the surface of the skin. The creation of biochemical sensors to track the composition of fluids changing during stress or exercise can be facilitated by these kinds of fabrics.^102^ The physiological reaction of the body to exercise and the kinematic elements of performance can be tracked with wearable sensors. Integrated sensors that are easy to use, comfortable to wear, and wearable are necessary to monitor physiological parameters in a natural way. The accessibility of this technology depends on textile-based sensors that work with textile manufacturing processes.

#### 5.2.2 Sweat and electrolyte monitoring

**Fig. 8:**
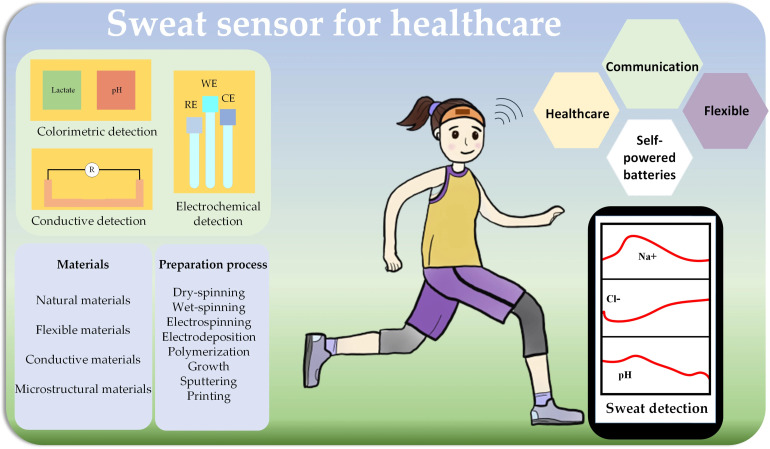
(a) Experimental set-up for sweat pH sensor. (b) Shirt to measure breathing rate using fabric stretch sensor. Published under the CC-BY lincense ref. 28 Copyright 2023, The authors. Published by MDPI, Basel, Switzerland.

The utility of a garment can be improved while preserving its typical tactile qualities by making the fabric itself the sensor.^28^ Rehydration has a key role in sports performance. Along with water, it is necessary to restore electrolytes lost through sweat.^103^ A cloth-based device has been created to analyze perspiration during exercise as part of the EU-funded Biotex project.^104^ A pH-sensitive indicator with a colorimetric response is used to dye a cloth channel. By employing a paired LED (light emitting diode) technique, LEDs above the channel are employed to detect color changes.^105^ With the use of moisture-wicking materials that are frequently found in sportswear, the method enables the direct collection and analysis of sweat in real-time (see Fig. 8). Real-time signal analysis makes it possible to provide the coach or player with prompt feedback.^106^

#### 5.2.3 Respiratory and breathing pattern sensors

A simple way to gauge how hard the body is working is to look at the way you breathe. Elevated heart rates are accompanied by rapid breathing, which indicates our perceived level of exertion. Breathing patterns across several training sessions could be measured using a wearable, non-intrusive sensor to evaluate exercise recovery and performance. Strain-based breathing sensors provide quicker reaction times but less biochemical information than biochemical sensors like sweat pH sensors. Conversely, optical sensors are highly accurate but frequently need external power sources. Combining these various sensing methods could strike a compromise between user comfort and real-time accuracy. Certain exercises, like yoga and pilates, also call for deliberate, calm breathing.^107^ Sports performance and exercise tolerance have been demonstrated to increase with respiratory muscle training in athletes.^108^ For such procedures, biofeedback from breathing rate and pattern measurements can be helpful.^109^ Additionally, it is helpful for overall health and well-being. Improved breathing practices are especially beneficial for people who are stressed out. One way to assess breathing rates is to use fabric strain or pressure sensors to detect the expansion and contraction of the ribcage.^110^

#### 5.2.4 Motion, posture, and advanced sensor materials

Building upon respiration monitoring approaches, modified foams coated with the conductive polymer polypyrrole (PPy) or loaded with carbon are placed within a pocket of an article of clothing or chest-strapped at the ribcage. When the rib cage stretches during inhalation, this compresses the foam and improves its conductivity in yoga and pilates.^111^ Moreover, motor skill training is based on dynamic postural alignment. Dynamic, flowing motions rather than static stances are the foundation of athletic movement. Good sports posture is a collection of postures that work together to create effective movement. The close-fitting nature of sports apparel makes it perfect for capturing the kinematics of an athlete.^112^ Giving the athlete feedback on this knowledge helps them become more conscious of their bodies, which could lead to better technique. The most popular method, which might not always be useful, is evaluation using only the human eye and video. While pricey camera equipment and close supervision, a coach might not be available, sports clothes are a universal requirement. CEA-Leti has created the sodium sensor. It is made of a solid contact ion-selective electrode (SC-ISE) and a gold reference electrode, and it is constructed on a flexible Kapton surface. It is composed of a polymeric membrane with a potential that varies with the concentration of salt covering a gold layer.^113^ Polypyrrole is the polymer utilized in this instance. Since the early 1990s, conducting polymer has been effectively utilized for solid contact ISEs. Other often utilized polymers are thiophene and aniline. While sportswear is a universal need, the doping material, which confers conductivity to the polymer, can also be altered to allow for a wider scope for optimizing sensor performance.^114^

## 6. Applications in sports performance enhancement

### 6.1 High-tech textiles for competitive sports

In worldwide professional sports, technology is as fundamental to success as essential components like nutrition, training, and mental toughness. Consequently, many athletes seek to outperform their competitors by donning the proper high-tech apparel. Compression clothing, smart textiles, and wearable technology are all included in the term “high-tech textiles”. Materials used to make smart textiles are those that have environmental adaptability.^115^ As an example, consider phase-change materials, which, depending on the surrounding temperature, can either cool or warm a material, or shear-sensitive materials that react to mechanical changes. Some antibacterial fabrics are developed, for instance, incorporating silk fibroin nanoparticles (SFNPs) and silver nanoparticles (AgNPs), to provide comfort to the wearer, which is also suitable for use in sports.^116^ In recent years, wearable technology has seen a great deal of research, and numerous cutting-edge solutions have been introduced to the market. Several studies have been conducted to demonstrate the benefits of compression clothing, particularly with regard to enhanced performance during training and recuperation.^117^ The physiological measures, such as heart rate, VO_2_ max, blood lactate concentration, and biomechanical values, such as muscle vibration and proprioception, were analyzed in order to assess the effect. The research setups of the utilized studies varied greatly.

Variations were evident based on the time the textiles were worn (during training, for regeneration, or for both), the number of test subjects, the athlete's professional status, the level of exercise intensity, and the metrics that were analyzed.^118^ In the research program of Gill *et al.*, the compression garments functioned as a tool for regeneration.^119^ According to some research, the athletes used compression clothing for both phases,^115^ either an endurance training session or a circuit specialized to team sports was used to test the compression fabrics. The outcomes greatly differed based on the extremely varied study setup. Athletes who used compression clothing showed improvements in their increased force, mean sprint time, and jump height, according to Dascombe *et al.*^120^ But some investigations contradicted these effects. The test subjects of Duffield *et al.* and Trenell *et al.* reported decreased soreness in their muscles when wearing compression garments. The benefits of biomechanical effects in smart materials were mainly investigated in swimming suits. While studies like Duffield *et al.* and Trenell *et al.* revealed minor biomechanical advantages,^121,122^ Chatard *et al.* and Gill *et al.* reported considerable improvements in recovery. These discrepancies imply that test design, exercise type, and garment pressure all have a significant impact on how effective compression wear is. In addition to selecting the right material, the structural makeup of the fabric is significant.^117,119^ According to Toussaint *et al.*, there are three types of water resistance that swimmers encounter: area, form, and friction resistance.^123^ As a result, researchers looked into how swimming suit design affected swimming kinematics and energy use. Different swimming suits with varying surface structures or lengths were employed for the test. A mechano-chemical coating was present on one of the wetsuits.^124^ In terms of the linear velocity and water resistance effect observed by the pelvis, the coating was beneficial. It is possible that Dantas *et al.* will also demonstrate an improvement in wetsuit water resistance. Both a physiological and a mechanical preventative impact can be achieved with functional fabrics.^125^ The ability of goalkeepers' pads to dampen was demonstrated by Schmitt *et al.* They might demonstrate that the cushioning is insufficient to protect the pelvis, and they might go on to create clever substitutes. Vital signs like blood pressure, body temperature, heart rate, and breathing sounds can now be directly measured using this technology without the need for an additional device.^126^ These features are currently mostly utilized in the fields of work protection and medicine. As a result, a large amount of research on this subject was discovered; a few will be briefly addressed below.^127^ Numerous papers discuss various approaches to incorporating wearable technology into textiles to measure important metrics. Movement analysis is very interesting, in addition to biofeedback and vital parameter monitoring.^128^ A few studies have been conducted using strain gauges and pressure sensors to identify movement in the human body after impact and release.^129^ Recently, a research group developed a pressure sensor by coating a woven cotton fabric with a polyacrylamide-LiCl ionic hydrogel, forming an integrated capacitive sensing network, which exhibits promise for wearable electronics.^130^ A suit for total body movement was created by Mazzoldi *et al.*^131^ Wearable technology is quite uncommon in the sports industry when compared to the health sector.^132^ Despite this, there are several cutting-edge goods on the market, such as the “miCoach” training system, which tracks exercise movements and intensity. Alternatively, a cutting-edge skiing coach created by TU Munich spinoff business Mocon.^115^ The skiing instructor is made up of a standard cell phone and an intelligent, pressure-sensitive sole.

### 6.2 Role of smart fibers and nanotextiles in performance enhancement

The term “smart fiber” describes fiber that, *via* the processing of smart materials, has the capacity to sense, react, and discover functions to the internal condition and external environment. The important traits and functions of the intelligent fiber system include sensing, feedback, information recognition and accumulation, response, self-diagnosis, self-repairing, and self-adjusting capabilities.^64^ The ability to perceive both the internal and external surroundings, as well as identify and detect external stimuli, including light, heat, stress, nuclear radiation, and variations in magnetic intensity, is known as the sensing function of the smart fiber.^133^ A fiber with a shape memory effect is referred to as a shape memory fiber. Shape memory fibers can revert to their original shape when they are exposed to specific external stimuli, like pressure and temperature. Shape memory metals, hydrogels, and polymers are the primary components of shape memory fibers. Shape memory polymers have advantages over shape memory alloys due to their low density, easy production, low cost, and high recoverable strain.^134^ These days, the creation of shape memory fibers in response to light, heat, stress, nuclear radiation, and magnetic intensity variations is receiving an increasing amount of interest. Most substances that change color when exposed to light are organic compounds that contain isomers. These isomers can change their configuration in a reversible way and change their hue, making them photosensitive. According to the discoloration mechanism, certain compounds will undergo reversible changes in their molecular structure or electronic energy levels when exposed to ultraviolet or visible light. Thus, the result in the formation of new compounds with different absorption spectra, and the compound will return to its original state when exposed to another light source.

A type of optical composite fiber called an optical fiber can contain light energy and transfer it in a waveguide mode. It has outstanding transmission performance and is also known as smart fiber. It is made up of two sections: the cladding and the core. Step type and gradient type are the two types of fiber architectures that can currently construct a waveguide transmission.

**Fig. 9:**
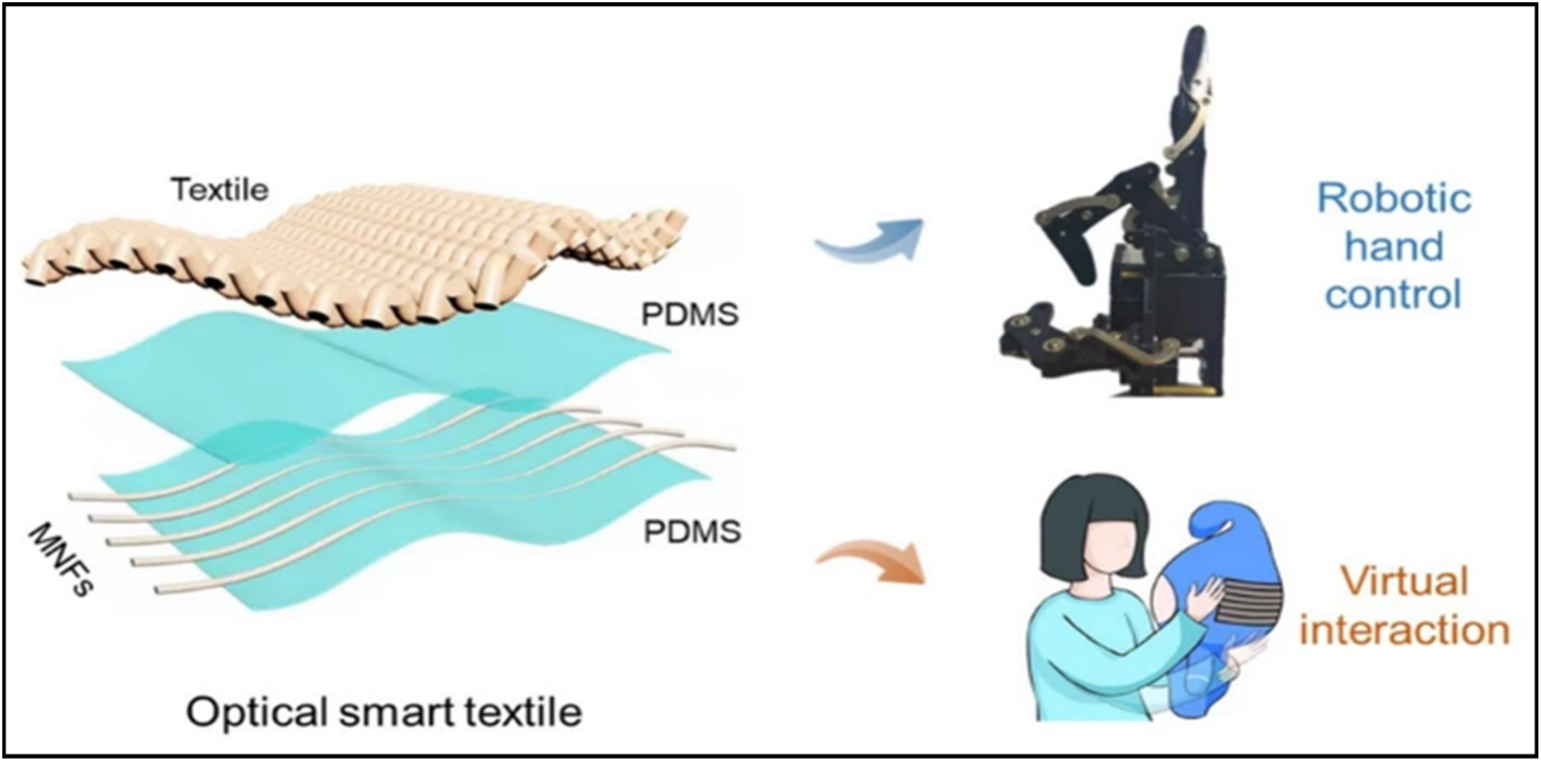
Optical micro/nano fibers enabled smart textiles Reproduced with permission^135^ Copyright 2022, Donghua University, Shanghai, China. Published by Springer Nature.

Optical fiber is frequently utilized as a sensing material because it can transmit and perceive information simultaneously. Currently, optical fiber sensors have highly developed application technology. By integration of a polydimethylsiloxane (PDMS) patch embedded with optical micro/nanofibers (MNF) array, an optically driven wearable human-interactive smart textile is proposed (see Fig. 9). These fibers allow for real-time sensing, signal transmission, and gesture recognition. The system provides seamless communication between the human body and electronic devices through highly sensitive pressure-dependent bending loss of MNF.^135^ Any fiber whose characteristics can reversibly change with temperature is said to be temperature sensitive. Research on temperature-sensitive discoloration fiber, moisturizing fiber, and heat preservation has been extensive. Dynamic fiber, such as the “Ventcool” fiber created by Mitsubishi Rayon Fiber Corporation, is able to crimp fast when conditions are dry and instantly stretch when there is a high level of humidity.^136^ A conductivity fiber is one that exhibits excellent electrical conductivity, can be disputed charge through electronic conduction and corona discharge, and has a specific resistance of less than 1 × 10^7^ Ω cm under standard conditions (20 °C, and 65% relative humidity).^137^ Static electricity is mostly employed today for electrical signal detection and transmission, electromagnetic wave absorption, and static electricity removal. Three categories can be used to categorize conductive fibers: inductive, ion, and electron. Hydrogels and aerogels made from MXene and various forms of nanocellulose can improve electrical and mechanical performance.^138^

Gel fibers that respond to changes in pH are known as pH-responsive gel fibers. These fibers alter in volume or form. At the molecular, macromolecular, and intermolecular levels, stimulus responsiveness is the basis for this alteration. Some wealthy nations are currently seeing significant advancements in the study of pH-responsive gel fiber. Moreover, fibers, such as impact-resistant fiber and antibacterial, deodorant fiber, are constantly created to sustain human health as awareness of safety protection increases. The most often utilized type of them is selective antibacterial fibers. Selective antibacterial fiber is a type of smart fiber that, by including an antibacterial chemical, may either inhibit or eliminate surface germs.^64^ On the surface of the skin, antibacterial fiber can keep some microbes growing and reproducing at a normal rate. Currently, the two primary ways of preparation for it are fiber alteration and finishing. Three types of antibacterial agents can be distinguished: composite antibacterial agents, inorganic antibacterial agents, and organic antibacterial agents. In the current textile and clothing industry, research hotspots include smart fibers, smart textiles, and their applications, as well as potential future development trends. High-tech functional textiles are based on smart textile materials. It can enhance living standards, raise the added value of goods, enhance working conditions, and satisfy industry demands. It will unavoidably become more prevalent in the application market in the future. Although it is still in its infancy and not yet fully developed, it represents a significant area of economic growth for the textile industry going forward.

## 7. Thermoregulation and biochemical monitoring

### 7.1 Importance of thermoregulation in sportswear

The mechanism by which the body controls its core temperature is thermoregulation. The body attempts to achieve a thermal steady state by balancing metabolic heat production and heat loss during activity in warm conditions, mostly through sweat evaporation.^139^ For endurance athletes in particular, thermoregulation is required because they are less able to regulate their body temperature in hot, muggy conditions, which raises the risk of heat illness and negatively affects performance.^140^ In scientific literature, techniques to improve bodily cooling have drawn a lot of attention.^141^

The 2021 summer olympics in Tokyo were among the hottest and most humid ever (averages of 32.2 °C and 70% relative humidity). Therefore, attention to the area is justified. Athletes can cool off with a variety of products, including ice slurries, neck coolers, cooling/ice vests, and cold beverages. However, due to practical limits such as excess weight, skin irritation/discomfort, and ingesting difficulties, these choices could be challenging to deliver during activity.^142^ In addition, due to laws and restrictions, several of these cooling alternatives are not practical to use during events like triathlons, long-distance cycling, and marathons. Choosing clothing plays a critical role in comfort, a complex sensation made up of various sensory inputs, including psychological, sensorial, and body-movement factors.^143^ It also presents a unique opportunity to aid thermoregulation during exercise in the heat without the practical limitations of the cooling strategies.^144^ Clothes naturally improve insulation, which in hot climates acts as a barrier against heat loss through evaporation. Sportswear, on the other hand, attempts to counteract this insulating effect by using lightweight, breathable designs made of synthetic materials that enhance moisture transport, or the wicking away of sweat, to encourage evaporative heat loss, maximize wearer comfort, and improve performance discomfort.^145^ Clothing choice is a significant factor in reducing the risk of heat stress when exercising in the heat, according to Sports Medicine Australia (SMA). According to SMA guidelines, the best ways to enhance sweat evaporation and heat dissipation are to remove superfluous garment layers, minimize skin cover, and wear lightweight, breathable clothing. But when it comes to some of the aspects of clothes, such as fabric composition, knit structure, and fit, these recommendations are vague.^146^ The lack of specificity could be the result of the inconsistent assessment parameters and methods applied in the various studies in this field, which have led to an insufficient amount of evidence.^147^

Research has looked at a range of materials and fabrics, such as natural and synthetic fibers, mixes of natural and synthetic fibers, and chemically treated fibers, to determine how well sportswear maximizes thermoregulation, comfort, and sports performance.^148^ Various levels of insulation, mass, and air permeability, together with various knit structures and fits (such as compression *versus* skin covering), are some of the material attributes that define the garment under examination.^149^ Sportswear is made of a variety of materials, such as synthetic fibers made by chemical synthesis (polyester, nylon) and natural fibers synthesized from plants and animals (such as cotton, and wool). Regarding comfort and thermoregulation, each fabric has advantages and disadvantages that vary depending on the material composition.^150^ Due to its inexpensive cost, silky feel, and dimensional stability, polyester is the most widely used synthetic material in sportswear. Its poor moisture absorption, however, may be a drawback in extreme sweating scenarios, as the extra moisture on the skin surface could provide uncomfortable and wet skin feelings.^151^ Sportswear made of polyester and cotton was compared by De Sousa *et al.* and Roberts *et al.* While exercising, Roberts *et al.* reported notable increases in comfort and thermal perception with polyester, and De Sousa *et al.* reported notable decreases in core temperature.^152,153^ Although both studies looked at identical textiles, Roberts *et al.* concentrated on subjective comfort, whereas De Sousa *et al.* stressed physiological temperature changes. The disparity in results demonstrates how evaluation standards and experimental setup affect the interpretation of the thermoregulatory performance. Nonetheless, Roberts *et al.*, testing parameters included mild, dry weather (20.6 °C, 47.5% RH, and low-to-moderate intensity workouts spaced out by 70 seconds of rest, which might not have caused excessive perspiration).^154^ Wearing an upper body compression garment made of nylon while exercising resulted in much less clothing wetness than when wearing cotton.^155^ Conversely, despite employing comparable testing procedures and identical fabric compositions, Leoz-Abaurrea *et al.*^156^ found no appreciable decreases in core temperature, skin temperature, sweat rate, or sweat loss when comparing sports clothing made of nylon and cotton.

### 7.2 Wearable technologies for biochemical analysis

**Fig. 10:**
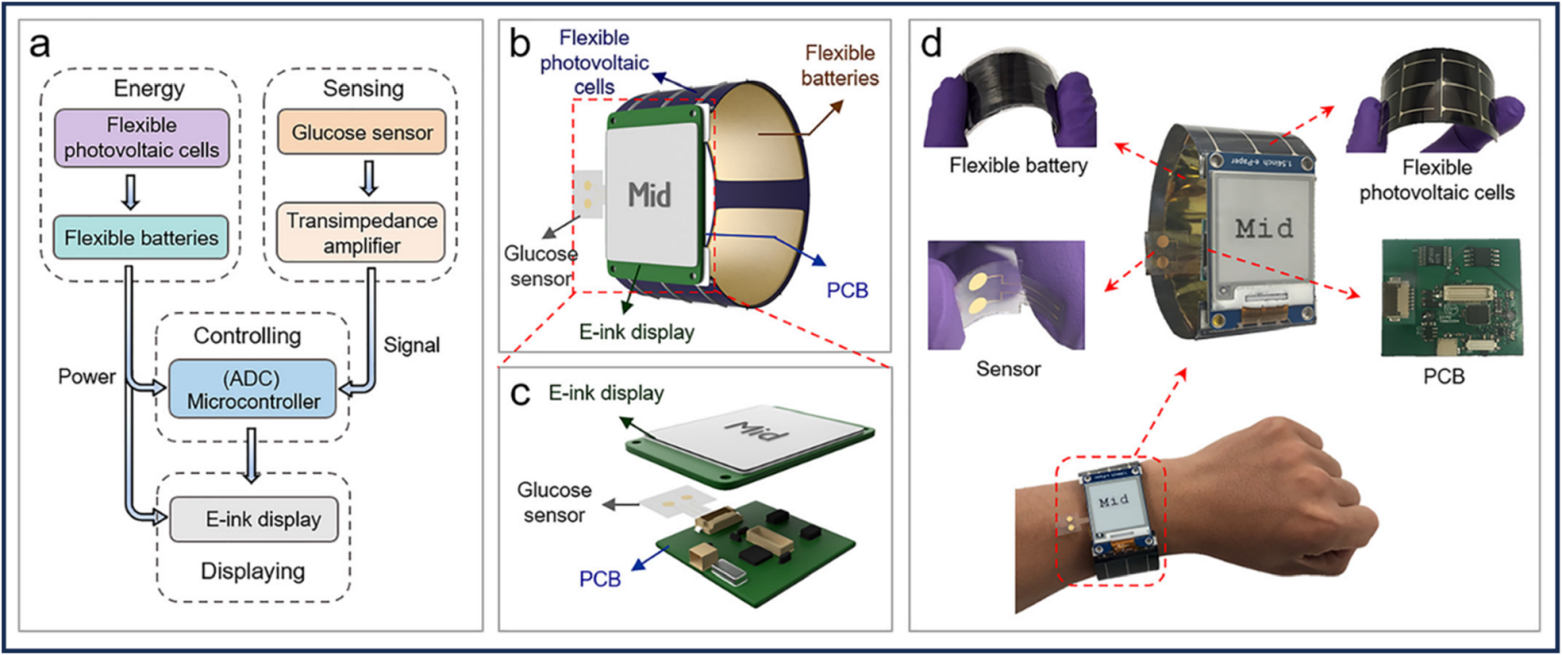
(a) System-level block diagram of the self-powered smartwatch. (b and c) Schematic illustrations of the self-powered smartwatch. (d) Images of the smartwatch on the wrist and separate components. Published under the CC-BY license^158^ Copyright 2019, The authors. Published by American Chemical Society.

Biochemical analysis was previously restricted to clinical and laboratory settings, but wearable technologies have rapidly improved to offer new opportunities. The way that biochemical markers are understood and managed is changing, thanks to these developments, which are making it possible to monitor health in real time and gain personalized insights into these markers. Typically, biochemical analysis of wearable technologies includes sensors that quantify different biomarkers in body fluids like saliva, perspiration, and interstitial fluid. These sensors provide important information about physiological and metabolic conditions by detecting and quantifying certain chemicals like glucose, lactate, and electrolytes using the concepts of electrochemistry, optics, and microfluidics. Especially for those with diabetes, one of the most prominent uses of wearable biochemical sensors is glucose monitoring. Wearables like continuous glucose monitors (CGMs) provide a less intrusive option to finger-prick blood tests, which are the traditional method of measuring glucose. To monitor the amount of glucose in the interstitial fluid, a tiny sensor is placed under the skin and used with CGMs.^157^ Users can better control their condition by using real-time glucose readings and trends provided by data that is transferred to a wearable device, like a smartwatch. One interesting development in wearable biochemical analysis is the use of sweat-based sensors. These sensors are frequently included in skin-attaching bands or patches. Sweat is analyzed to determine the levels of several biomarkers, such as glucose, lactate, and salt. For example, during exercise, a sweat sensor may measure the amount of lactate produced, giving information on the performance of an athlete and recovery requirements. A fully integrated, self-powered smartwatch for real-time sweat glucose monitoring has been developed, which uses flexible glucose sensors, Zn–MnO_2_ rechargeable batteries, and photovoltaic cells to enable continuous operation without external power (see Fig. 10).^158^ The non-invasive method provides an ongoing flow of data that can be utilized to customize training plans and enhance sports performance. Additionally, hormones, electrolytes, and other biochemical indicators associated with stress, hydration, and general health can all be measured by saliva sensors. These sensors are being investigated for their potential to offer non-invasive information on a variety of health issues, although they are less common than sweat sensors.

Various technologies are employed by wearable biochemical sensors to identify biomarkers. Electrochemical sensors consist of electrodes that react with compounds present in the body fluid, producing an electrical signal that is directly proportional to the quantity of substance. In contrast, optical sensors employ light to identify alterations in the fluid characteristics or to gauge absorbance or fluorescence linked to certain biomarkers. For health management, Bio-Tex (biosensing textile) aims to create specialized biochemical sensing methods to use sensors spaced over a cloth to track bodily fluids. The first applications are expected to be for personal sports performance and training, and health. There is a platform built on textiles for the gathering and sending of perspiration samples generated during workouts colorimetric pH sensor.^159^ It is necessary to create a procedure for sample collection, delivery to the sensor location, and removal to perform a real-time biochemical analysis of sweat. Textiles having built-in moisture-wicking capabilities can be used to create such a fluid management system for wearable sensors by capillary action. Wearable Technology for Bio-Chemical Analysis of Body Fluids: A fabric patch is prepared and then, sweat is collected from the skin surface during exercise and sent to the sensing area *via* a pre-established route. For this reason, a commercial polyimide/lycra® blend, provided by Sofileta, that is frequently used in sportswear, has been selected by one study.^114^ The study found that sweat rate and component concentrations vary throughout the surface of the skin. As a result, it is essential to always apply the patch in the same spot. For convenience of access, the upper body is recommended. The hands, lower back, scapula, forehead, and chest are among the regions with high perspiration rates. The latter choices are the better options because the patch can be attached to a belt that is worn while working out.^160^ There are various difficulties and potential directions for wearable biochemical technologies as they develop. Constant work is being done to improve wearable device lifespan, user comfort, and sensor accuracy and reliability. It is also critical to address privacy issues regarding the private health data these gadgets gather. As these technologies proliferate, it will become increasingly crucial to guarantee data security and regulatory compliance.

## 8. Challenges and opportunities

### 8.1 Current challenges in the development and application of smart textiles

Some of the challenges that skilled textile designers must overcome are covered in the following section. Considering some of the needs that textile circuits must meet may help you appreciate the difficulties that researchers hoping to create smart textiles must overcome. During the manufacturing process and when smart textiles are used in everyday life (*e.g.*, by wearing them as clothing), circuits must withstand harsh mechanical conditions. The smart textile should be durable enough to withstand daily use without compromising its comfort or washability due to the absence of circuitry. Washability and user comfort are the main issues with sports-focused smart textiles, as opposed to medical wearables, where miniaturization and sterilization predominate. Lightweight and large-capacity power sources are necessary for circuits to assure several hours (or more, depending on the intended end-user application) of independent operation. Commercial smart textiles must adhere to regulations from the electronics and textile industries. These requirements are frequently extremely strict and sometimes even conflicting. These are a few major obstacles that smart textile development must overcome.

#### 8.1.1 Mechanical environment

Smart textile fibers may be subjected to substantial tensile strains and bending radii significantly less than 1 mm, in contrast to flexible display systems that are designed to be rolled around cylinders with diameters of a few cm.^161^ The location of the textile circuit on the body and the textile architecture determine how much tensile strain e-fibers are subjected to during dynamic wear conditions.^162^ The top back of shirts and jackets is where textile fibers undergo the most tension. Textile simulations have revealed that strain on a shirt at the shoulder blades can reach 20%.

#### 8.1.2 Washability

The wearer of early commercial smart textiles, like the Phillips and Levi-developed ICD textile coat, had to take out all electrical parts, including the wire, before they could be washed.^163^ This is one of the major challenges that smart textiles are facing, which industries need to overcome for better products.

#### 8.1.3 Power supplies

Conventional rechargeable batteries power the majority of smart textiles; however, they are big, bulky, and difficult to completely integrate with the textile architecture.^50^ The development of lightweight, alternative conformal power-generating and storage systems is highly motivated. Supercapacitors, solar cells, elastic or flexible batteries, and energy-harvesting gadgets like piezo and thermo generators are a few examples.^164^ Regretfully, in terms of maximum current and capacity, none of these devices can compete with conventional batteries. In order to enhance the functionality of these textile-compatible power supplies for upcoming smart textile applications, immediate research is required.

#### 8.1.4 Development and marketing of new products

The unequal contributions from the apparel and electronics sectors lead to applications that are not fully integrated. Since the electronics industry still dominates product development, most research efforts are directed toward finding technical solutions to challenges like resolving washability issues or integrating microchips and computer systems into garments. The apparel industry still sees very few application advancements that take into account or incorporate the unique product creation and processing procedures used.^165^ It is challenging to fully integrate electronics and fashion because of this unequal contribution, and smart textiles have an even harder time standing out from both traditional apparel and current electrical equipment. Textile scientists, polymer chemists, physicists, bioengineers, software engineers, consumer experts, and fashion designers are among the multidisciplinary professionals who will be needed for successful design and development. Organizing a shared gathering and deciphering the technical terms used in each sector might be difficult. Moreover, there is a dearth of a unified vision for the development of smart textiles across many institutions and research facilities. Product development is frequently an expensive and pointless undertaking.

### 8.2 Future opportunities and potential developments

According to growing customer expectations for creative solutions and technological improvements, the future of smart textiles is full of possibilities. Wearable sensors incorporated into textiles provide the potential to revolutionize the healthcare industry through smart textiles. These textiles can continuously give patients and healthcare professionals health data by monitoring vital signs, including blood pressure, heart rate, and glucose levels in real-time. This may result in early identification of health problems, more proactive management of chronic illnesses, and individualized treatment regimens. Additionally, patient care may be improved by smart textiles with therapeutic qualities, such as those that administer medication or physical therapy. Smart textiles provide enhanced performance monitoring and injury prevention in the sports and fitness domain. Wearable clothing with built-in sensors can monitor body temperature, muscle activity, and biomechanical movements to give athletes comprehensive information about their physical and performance states. By using the mentioned data, training plans may be optimized, techniques can be improved, and the chance of injury can be decreased. Furthermore, intelligent textiles with built-in heating or cooling components can assist in controlling body temperature, improving comfort during vigorous exercise. Electronics are finding their way into fashion, opening new possibilities for dynamic and interactive apparel. Intelligent fabrics can modify their hue or design in reaction to external cues or user input, providing a personalized and flexible fashion experience. Moreover, clothes with integrated LEDs or touch-sensitive controls can offer people new opportunities to show their individuality and engage with their apparel. Both safety and environmental monitoring may benefit from smart fabrics. Sensor-enabled fabrics can identify allergens, pollutants, or dangerous gases and notify the wearer in real-time. The feature could improve the safety and well-being of people who operate in high-risk areas, such as first responders or industrial workers. Smart textiles may improve the functionality of equipment and uniforms used in military applications. The operational efficacy and military safety could be enhanced by features like environmental sensors, adaptive camouflage, and integrated communication systems. Smart textiles promise to improve healthcare, sports, fashion, environmental monitoring, and more in the future through a combination of technology innovation and real-world application. Future generations of E-textiles are anticipated to be more autonomous than current models, incorporating energy harvesting techniques that surpass the constrained battery-based configurations of today. However, a major obstacle is still reaching scale on par with conventional clothing manufacturing. The assimilation of smart textiles into daily life is anticipated to become more impactful and smoother with continued research and development.

## Conclusions

The paper investigates the latest trends in smart textiles and wearable technologies designed for sports and examines their impact on performance and comfort. Furthermore, the use of smart fibers and nano textiles has helped advance performance, especially in the case of sports. In addition, smart textiles have sensors and other electronic devices that can examine parameters including heart rate, temperature, and muscle activity, which eventually helps to enhance the performance of athletes. The importance of wearable electronics and textile sensors, in performance enhancement through monitoring thermoregulation and biochemical assessment in the sports field, has also been recognized. On the other hand, several factors such as technical barriers, cost restrictions, importing solutions in the context of today, and other factors challenge the development and use of the smart textile, respectively. Despite a few challenges, smart textiles and wearable electronics open the doors for maximal benefits in the sports sector. An extensive overview of the current state and advancement of smart textiles for sports has been given in this paper, and it has been pointed out that more research must be done.

## Author contributions

Md Touhidul Islam and Md Imran Hosen have contributed to the conceptualization, resources, methodology, visualization, data collection, original draft writing, and editing; Md. Abdullah Al Mamun has contributed to formal analysis, editing, and reviewing; Tariful Islam has contributed to the editing and reviewing of this manuscript; Tarikul Islam has contributed to conceptualization, investigation, writing the original draft, reviewing, editing, validation, visualizations, and supervising at all stages of preparing the manuscript.

## Conflicts of interest

The authors declared no conflict of interest.

## Abbreviations

AgNPs: Silver Nanoparticles
AI: Artificial Intelligence
ArDiff: Area Difference
BP: Blood Pressure
CAGR: Compound Annual Growth Rate
CGM: Continuous Glucose Monitor
CVD: Cardiovascular Disease
ECG: Electrocardiogram
EU: European Union
HR: Heart Rate
HRV: Heart Rate Variability
i-Cool: Integrated Cooling
ICP: Intrinsically Conducting Polymer
IDC: International Data Corporation
IMU: Inertial Measurement Unit
IR: Infrared
ISE: Ion Selective Electrode
LED: Light-Emitting Diode
MaxCorr: Maximum Correlation Coefficient
MNF: Micro/Nanofibers
mHealth: Mobile Health
NiTi: Nickel Titanium
PDMS: Polydimethylsiloxane
pH: Potential of Hydrogen
POF: Plastic Optical Fiber
PPy: Polypyrrole
PVC: Polyvinyl Chloride
PVDF: Polyvinylidene Fluoride
RH: Relative Humidity
RR: Respiratory Rate
SC-ISE: Solid Contact Ion-Selective Electrode
SFNPs: Silk Fibroin Nanoparticles
SMA: Shape Memory Alloy
SMA: Sports Medicine Australia
Smart-NT: Smart Nonwoven Textile
UK: United Kingdom
USD: United States Dollar
UV: Ultraviolet

## Data Availability

No primary research results, software or code have been included and no new data were generated or analysed as part of this review.
